# A Randomized Clinical Study Comparing Aesthetic, Functional and Sensory Outcomes of Distant Pedicled Flap Versus Free Flap Reconstruction of Upper Limb Defects

**DOI:** 10.1055/s-0046-1816055

**Published:** 2026-02-16

**Authors:** Saradha Ramani, Prakash Chandra Kala, Pawan Kumar Dixit, Deepti Katrolia, Shilpi Karmakar

**Affiliations:** 1 Department of Burns and Plastic Surgery, All India Institute of Medical Sciences, Jodhpur, Rajasthan, India

**Keywords:** upper extremity, wounds, microsurgery, reconstructive surgery

## Abstract

**Background:**

Upper limb tissue defects may result from trauma, infection, burns, or tumor resection. Small superficial defects are amenable to skin grafting, whereas larger full-thickness defects with a wide zone of injury require coverage with pedicled or free flaps. The goal of reconstruction in upper limb defects is not only to achieve coverage but also to restore motor and sensory function as well as aesthetics.

**Materials and Methods:**

In this randomized study, patients who underwent resurfacing of upper limb defects between July 2022 and June 2024 were divided into two groups: distant pedicled flap (Group 1) and free flap (Group 2). Aesthetic, sensory, and functional assessment were performed at 3 and 6 months postoperatively, and statistical significance was analyzed between the two groups.

**Results:**

A total of 40 patients were enrolled, with 22 patients in the pedicled flap group and 18 in the free flap group. Aesthetic analysis using the Likert scale showed better results in the free flap group. Both subjective and objective functional assessment showed better results in the free flap group at 3 months, with comparable results in both groups at 6 months. Sensory assessment showed similar results in both groups.

**Conclusion:**

With free tissue transfer, better overall outcomes were observed, including improved aesthetic appearance, earlier functional recovery, earlier return to work and daily activities, and better quality of life. In the presence of a dedicated microsurgical team, better reconstructive goals can be accomplished in upper limb reconstruction.

## Introduction

Upper limb tissue soft defects may occur following trauma, infection, burns, or tumor resection. Structures such as blood vessels, muscles, tendons, nerves, and bones may be exposed, and multiple surfaces can be involved. 1 Small superficial defects are amenable to reconstruction with skin grafts. However, larger, full-thickness complex defects with wide zones of injury and questionable viability of adjacent tissues require proper debridement and coverage with flaps. Moreover, the goal of surgical reconstruction is not just to cover the defect but also to restore motor and sensory function as well as aesthetics. 2

Flaps used for resurfacing complex upper limb defects include pedicled flaps (locoregional and distant) and free flaps. Locoregional flaps are limited to the resurfacing of smaller defects of limited donor tissue is availability due to the shape and size of the upper limb, proximity to the zone of trauma, restricted mobility and limited reach of the flaps and the need for grafting of the donor site, giving an unsightly appearance. 3 Therefore, medium and large defects require coverage with either distant pedicled flaps or free flaps. Resurfacing with distant pedicled flaps typically involves multi-stage surgery, including reconstruction and subsequent flap debulking. Over the past five to six decades, improvements in microsurgical expertise and instrumentation have given rise to a wide range of reconstructive microsurgical options that circumvent numerous phases while achieving optimal coverage and function. 4

Various studies have been published so far evaluating the outcomes of pedicled flaps and free flaps individually. However, very few prospective studies provide clear, measurable outcomes in the form of objective scores. 5 6 7 8 Moreover, there is a paucity of studies comparing the outcomes of distant pedicled flaps and free flaps in upper limb reconstruction.

Hence, this study was planned to evaluate and compare the aesthetic, functional, and sensory outcomes of the two commonly used flap types—distant pedicled flaps and free flaps—in upper extremity reconstruction.

## Materials and Methods

This single-center randomized clinical study was conducted at our hospital from July 2022 to June 2024. A total 40 patients scheduled for resurfacing of upper limb defects were included in the study and randomized into two groups − distant pedicled flap (Group 1) and free flap (Group 2) with the help of a computer-generated table of random numbers.

Patients with soft tissue defects of the upper limb due to trauma, infection, soft tissue tumors, burns (thermal/electric) with exposed vital structures, and post-burn contractures were included in the study. Patients unfit for prolonged surgery or anesthesia or those unwilling to adhere to prolonged follow-up, and patients under 12 years of age were excluded. Ethical approval was obtained from the institutional ethics committee.

The surgery was performed under tourniquet control and loupe magnification (3.5–4.0 ×). The defect was created after adequate debridement. In the distant pedicled flap group, the flap was planned in reverse by bringing the hand to the abdomen. The flap was planned and marked as an axial flap based on a designated vessel or a random pattern flap, depending on the dimensions and location of the wound. The flap was raised, and insetting was done after proper hemostasis. Flap division and final insetting were performed at a second stage after 3 weeks.

In the free flap group, flap perforators were marked over the donor site using a handheld Doppler. The incision was deepened until the fascia and significant perforators were found. After the perforators were located, the source artery was traced and delineated. The incision was completed around the flap margin, and the pedicle was divided. Based on the state of the recipient vessel, end-to-side or end-to-end microvascular anastomosis of the pedicle artery and vein was performed with the upper limb recipient vessels. Inset of the flap was completed. In both groups, depending on the size of the donor defect, it was either closed primarily or resurfaced with a split thickness skin graft (STSG).

Demographic data and pre-and postoperative parameters were recorded, including etiology, site and size of the defect, surgery duration, flap size, and complications. Postoperatively, patients were followed up at 3 and 6 months, and aesthetic, functional, and sensory evaluations were performed. Statistical significance was analyzed between the two groups.

The overall aesthetic appearance of the flap was assessed using a 5-point Likert scale. Patients and an independent observer (plastic surgeon) were asked to rate the reconstruction on a scale of 1 to 5 using a questionnaire based on color match, contour, and texture. 9 The patient and an independent observer assessed the recipient site scar using the Patient and Observer Scar Assessment Scale (POSAS). 10 Assessment of disability was done using the QuickDASH Questionnaire. 11 The passive range of motion of all joints of the upper limb was measured using a goniometer to assess joint stiffness and to determine whether flap bulkiness or contracture inhibited joint movement. Power grip was assessed using a dynamometer and compared with the normal side. The grip strength in the affected limb was compared with that of the contralateral normal limb, and the result was expressed as a percentage.

Sensory recovery over the reconstructed flap was assessed and graded from 0 to 3+ based on the approximate surface area of the flap that was sensate 12 ( Table 1 ). Protective sensation was assessed using 10 g monofilament, as described by Semmes and Weinstein. Hot and cold temperature sensations were tested using the hot and cold test tube method. Static 2-point discrimination, as described by Webber, was measured using Disk-criminator. Assessment of two-point discrimination (2PD) was done only if the protective sensation was graded 2+ or higher.

**Table 1:** Grading of return of sensation in flaps

Grade	Perception of sensation
0	Flap completely insensate
1+	Sensation in <one-third of the flap
2+	Sensation in approximately two-third of the flap
3+	Sensation throughout the entire flap

Statistical Analysis was performed using IBM-SPSS version 21.0 (IBM-SPSS Science Inc., Chicago, Illinois, United States). Data was presented as mean ± standard deviation, frequency, and percentage. Continuous variables were compared using the unpaired *t* -test, and categorical variables were compared using the Pearson Chi-square test. Significance was defined as a *p* -value of less than 0.05 using a two-tailed test.

## Results

### Demographic Data

The mean age of patients included in this study was 38.85 ± 17.85 years, with the highest number of patients (35%) in the 21 to 39 age group. Trauma was the most common etiology, accounting for 86.4% of cases in the pedicled flap group and 61.1% in the free flap group. The demographic data of patients in both groups is shown in Table 2 .

**Table 2:** Demographic data of patients

	Group 1 (Pedicled flap)	Group 2 (Free flap)
No of patients	22	18
Mean age	38.09 ± 15.99 y	39.78 ± 20.34 y
Male/Female	16/6	13/5
**Etiology:**		
Trauma	19	11
Tumor	2	5
Infection	1	0
Post burn contracture	0	2

**Fig. 1:**
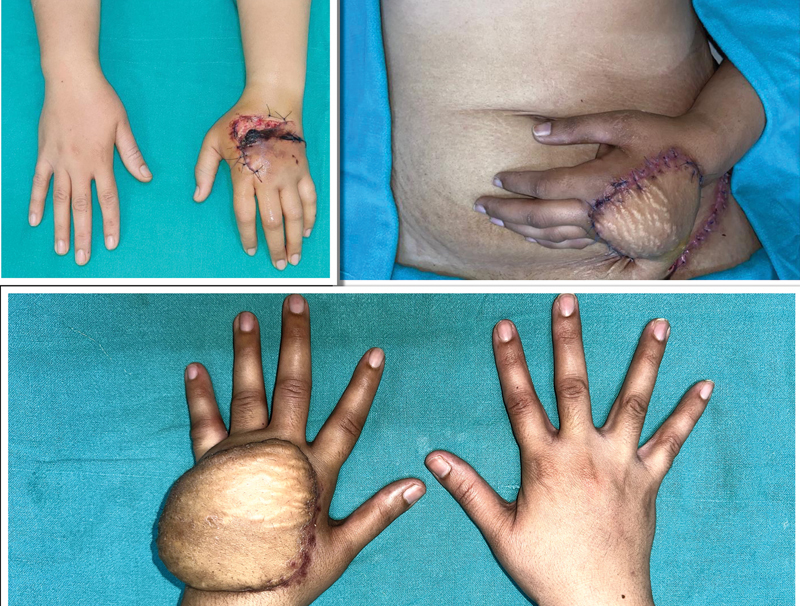
Shows pre- and postoperative images of a groin flap performed for a degloving injury over the dorsum of the hand.

**Fig. 2:**
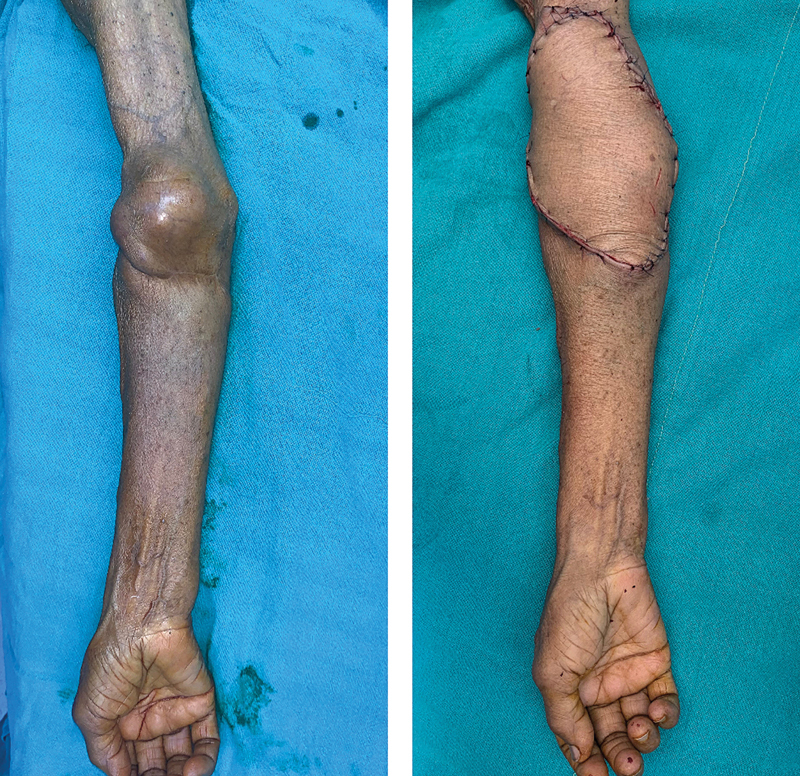
Shows pre- and postoperative images of a free ALT flap performed for a case of soft tissue sarcoma over the elbow. ALT, anterolateral thigh flap.

Fig. 1 shows pre- and postoperative images of a groin flap performed for degloving injury over the dorsum of hand. Fig. 2 shows pre- and postoperative images of free ALT flap performed for a case of soft tissue sarcoma over the elbow.

### Defect and Flap Characteristics

**Fig. 3:**
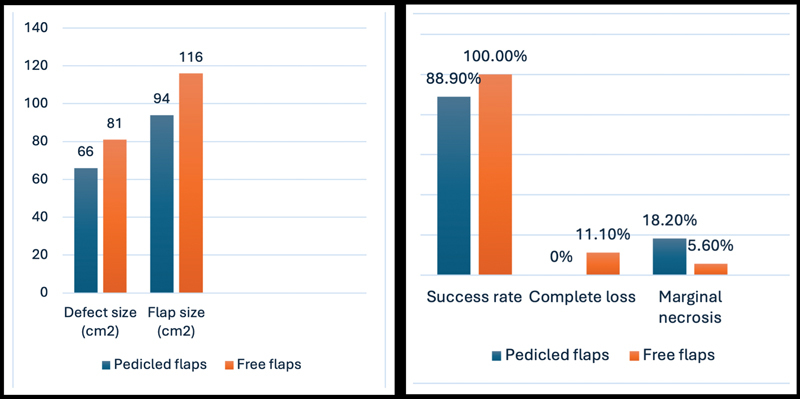
Shows defect and flap characteristics.

Forearm defects accounted for 35% of the cases. The mean defect size in this study was 72 ± 27 cm ^2^ , and the mean flap size was 104 ± 36 cm ^2^ . Defect and flap characteristics are presented in Table 3 . The donor site was closed primarily in 86.4% of pedicled flaps and 66.7% of free flaps. STSG were used to resurface the donor site in 13.6% of pedicled flaps and 33.3% of free flaps. Fig. 3 shows a comparison of defect and flap characteristics between the two groups.

**Table 3:** Defect and flap characteristics in the two groups

	Group 1 (Pedicled flap)	Group 2 (Free flap)
Anatomical region of defect:		
Forearm	6	8
Dorsum of hand	9	4
Palm	5	4
Elbow	2	2
**Flap performed:**	22 Groin flap: 11 Abdomen flap: 5 Thoracoumbilical flap: 5 Hypogastric flap: 1	18 Free ALT flap: 17 SCIP flap: 1
Mean defect size	66 ± 30 cm ^2^	81 ± 22 cm ^2^
Mean flap size	94 ± 32 cm ^2^	116 ± 38 cm ^2^

### Complications

There were no cases of complete flap loss in the pedicled flap group; however 18.2% (four cases) experienced marginal necrosis, which necessitated advancement and re-insetting of the flap in 2 cases and debridement of the discolored margin followed by grafting in two cases. In the free flap groups, 11.1% (two cases) experienced complete flap loss, and 5.6% (two cases) had marginal necrosis. In the two cases of complete flap loss, the free flap was debrided, and a pedicled flap was performed. The overall success rate was 100% in the pedicled flap group and 88.9% in the free flap group.

### Aesthetic Outcome

Table 4 summarizes the results of the aesthetic assessment performed by patients and observers using a 5-point Likert scale and the POSAS questionnaire at 3 and 6 months postoperatively.

**Table 4:** Aesthetic assessment at 3- and 6-mo postoperative

Parameter assessed	Group 1 (Pedicled flap)	Group 2 (Free flap)	*p* -Value
Mean Likert score (patient):			
3 mo	3.55 ± 0.60	4.01 ± 0.68	0.028
6 mo	4.18 ± 0.34	4.69 ± 0.48	0.0004
Mean Likert score (observer):			
3 mo	2.86 ± 0.64	3.56 ± 0.73	0.003
6 mo	3.32 ± 0.65	3.88 ± 0.81	0.024
Mean POSAS score (patient):			
3 mo	11.64 ± 5.84	11.69 ± 5.22	0.978
6 mo	9.91 ± 4.74	10.81 ± 4.71	0.564
Mean POSAS score (observer):			
3 mo	13.45 ± 8.24	11.44 ± 5.24	0.396
6 mo	11.91 ± 7.33	10.69 ± 5.11	0.571

At both 3 and 6 months postoperatively, significantly better mean patient Likert scores were observed in the free flap group, with *p* -values of 0.028 and 0.0004, respectively. Similarly, mean observer Likert scores were also significantly better in the free flap group. Recipient site scar assessment using POSAS score, as evaluated by both patient and observers, showed no significant difference between the two groups at both 3 or 6 months postoperatively.

### Functional Assessment

**Supplementary Video S1** Range of motion at 3 months postoperative in a case of groin flap for defect over dorsum of left hand.


**Supplementary Video S2** Range of motion at 3 months postoperative in a case of free ALT flap for left elbow defect. ALT, anterolateral thigh flap.


Table 5 summarizes the functional and sensory assessments of both groups of patients at 3 and 6 months postoperatively.

**Table 5:** Functional assessment at 3 mo and 6 mo postoperative

Parameter		Group 1 (Pedicled flap)	Group 2 (Free flap)	*p* -Value
**Mean Quick DASH score**	3 mo	24.18 ± 11.44	12.51 ± 6.29	0.001
	6 mo	15.68 ± 9.03	11.51 ± 5.91	0.116
**Range of motion (degrees):**				
Elbow flexion	3 mo	137 ± 6	142 ± 4	0.012
	6 mo	141.5 ± 3.78	142.50 ± 3.65	0.403
Wrist flexion	3 mo	47.38 ± 2.56	47.81 ± 3.15	0.648
	6 mo	50.5 ± 3.20	51.25 ± 2.24	0.406
Wrist extension	3 mo	45.48 ± 6.31	47.19 ± 6.05	0.411
	6 mo	51.1 ± 31.5	52.5 ± 2.58	0.138
MCP joint flexion	3 mo	73.18 ± 6.46	83.44 ± 4.37	<0.0001
	6 mo	82.5 ± 4.06	84.38 ± 2.50	0.094
PIP joint flexion	3 mo	85.68 ± 4.95	97.19 ± 5.15	<0.0001
	6 mo	96.3 ± 5.88	97.19 ± 5.15	0.617
DIP joint flexion	3 mo	66.82 ± 5.24	73.50 ± 2.45	<0.0001
	6 mo	73.1 ± 3.61	74.44 ± 1.75	0.158
**Grip strength**	3 mo	79.50 ± 8.50	90.38 ± 2.70	<0.0001
	6 mo	86.73 ± 4.48	80.72 ± 2.44	0.350
**Sensory assessment:**				
Protective sensation	3 mo	0.33 ± 0.48	0.41 ± 0.51	0.629
	6 mo	1.77 ± 0.87	1.82 ± 0.95	0.863
Temperature perception	3 mo	0.67 ± 0.48	0.47 ± 0.51	0.235
	6 mo	1.68 ± 0.48	1.47 ± 0.62	0.238
Two point discrimination	6 mo	29 ± 5	28 ± 5	0.574

The mean Quick DASH scores were significantly better in the free flap group at 3 months with a *p* -value of 0.001. At 3 months, a significant differences in passive range of motion of joints were seen in elbow flexion ( *p* = 0.012), metacarpophalangeal joint flexion ( *p* <0.0001), proximal interphalangeal joint flexion ( *p* <0.0001), and distal interphalangeal joint flexion ( *p* = 0.0001). Grip strength at 3 months was significantly better in the free flap group than in the pedicled flap group( *p* <0.0001). At 6 months, no significant differences in mean QuickDASH scores, grip strength or passive range of motion (ROM) were observed between the two groups. Supplementary Videos 1 and 2 show the range of motion at 3 months postoperatively in a patient of groin flap reconstruction and ALT flap reconstruction respectively.

**Fig. 4:**
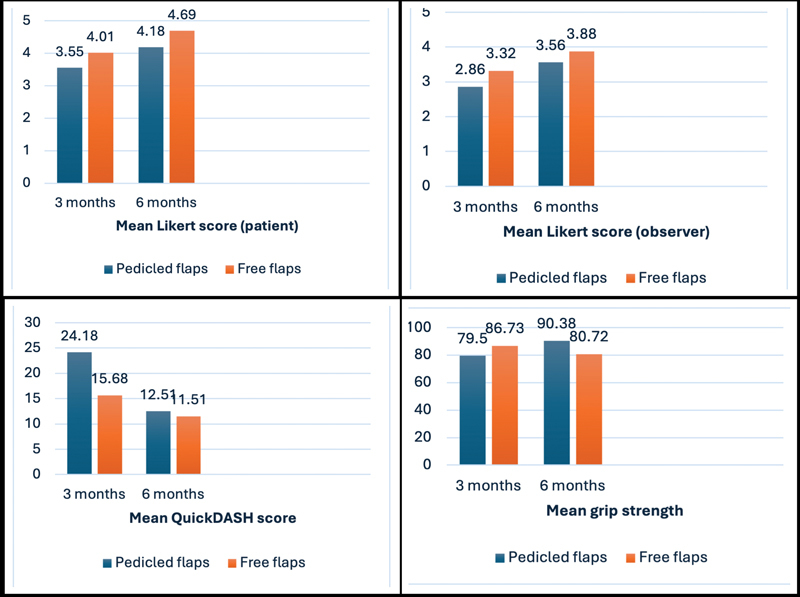
Depicts results of aesthetic and functional assessment in both flap groups.

There was no significant difference in sensory recovery between both groups at 3 and 6 months. The 2PD was not recorded at 3 months in any of the flaps, as none of the flaps met the criteria of 2+ or greater protective sensation. At 6 months, the mean 2PD values showed no significant difference between the two groups ( *p* = 0.574). Fig. 4 compares the aesthetic and functional assessment results between the two flap groups.

## Discussion

The field of reconstructive surgery has continuously evolved with the expansion of various flap options, including random pattern flaps, axial pattern flaps, and microsurgical free flaps. Given the unique nature of the upper limb in terms of its specialized function and developments in microsurgery, the concept of “reconstructive elevator” proposed by Gottlieb and Krieger has replaced the concept of “reconstructive ladder.” 13

There are well-known disadvantages with distant pedicled flaps, such as the need for second surgery for flap division, prolonged hospital admissions adding to the cost of treatment, added discomfort for the patient due to immobilization of the hand to the abdomen, difficulty in positioning in the presence of external fixators, the need for secondary surgeries for debulking or tendon reconstruction (in case of tendon injury) and joint stiffness necessitating multiple physiotherapy sessions. Despite various drawbacks, these flaps are easy and faster to raise, have a shorter learning curve, and no additional microsurgical and technical skills are required. 4

Free tissue transfer combines skin coverage with muscle, fascia, bone, and tendons making it ideal for the reconstruction of complex defects. The patient has no added discomfort of the limb attached to the abdomen. There is potential for flap thinning and sensory reinnervation of the flap in the same sitting. Single-stage complete reconstruction provides for early initiation of physiotherapy, thereby reducing joint stiffness and fibrosis. Though single-stage coverage is possible with free flaps, they demand longer operating time, better facilities, and a steeper learning curve, and there is a potential risk of flap failure with variable survival rates. 4

Considering the various advantages and disadvantages of both reconstructive options, we evaluated and compared the aesthetic, functional, and sensory outcomes of both flap groups subjectively and objectively.

In this study, the mean age of the patients was 38.85 ± 17.85 years, ranging from 17 to 70 years. A majority of patients (57.5%) were 21 to 59 years old, indicating the importance of reconstruction in the leading economically productive age group. The mean defect and flap sizes were comparable between the two age groups. No significant differences were observed in the management of the donor site in both groups, with the majority of the donor defects being closed primarily and the remaining being grafted.

The overall success rate in the pedicled flap group was 100%, and the free flap group was 88.9%. The complication rates observed in our study were similar and comparable to previously published individual studies on free flaps and pedicled flaps. Observations from our study, as well as from other studies 7 8 14 15 indicate that microsurgical failure in free flaps can lead to complete flap loss, whereas complete flap loss is less common in pedicled flaps, with marginal necrosis seen more commonly.

In terms of aesthetic assessment, significantly better mean Likert scores in the free flap group at 3 and 6 months (both the patient and the observer) show that aesthetically superior reconstruction − in terms of better color match, contour, and patient satisfaction − is possible with free flaps compared with pedicled flaps.

Subjective assessment of disability was measured with the Quick DASH questionnaire, while objective assessment of functional recovery was assessed by grip strength and passive ROM of all joints of the affected limb. Significantly better mean Quick DASH scores, grip strength, and range of motion were observed in the free flap group at 3 months, whereas the results were comparable between the two groups at 6 months. The observation of better range of motion, grip strength and lower disability in the free flap group at 3 months postoperatively points toward earlier functional recovery in the free flap group, likely due to single-stage comprehensive reconstruction permitting early mobilization and better freedom of movement for early initiation of physiotherapy thereby reducing tendon adhesions and joint stiffness. Due to the added discomfort of having the hand attached to the abdomen for 3 weeks in the pedicled flap group − which impedes proper mobilization and physiotherapy of joints − the range of motion of joints and grip strength were less compared with the free flap group at 3 months likely due to joint stiffness. Following proper physiotherapy, no significant differences were observed between the two groups at 6 months postoperatively.

In terms of sensory assessment, the scores for protective sensation, temperature perception, and static 2PD were comparable in both flap groups at 3 and 6 months, as no additional sensory nerve coaptation was done in either group to make the flap sensate and the flaps depended on spontaneous sensory reinnervation to regain sensation. The pattern of spontaneous sensory reinnervation of flaps observed in our study was in accordance with previously published studies on spontaneous sensory recovery of non-innervated flaps. 16 17

To the best of our knowledge, no previous comparative studies have evaluated the two groups of flaps. Hence, this study is a first of its kind comparing aesthetic, functional and sensory outcomes between the two flap groups using established objective scoring systems. This study also had certain limitations. The sample size was small, with an unequal number of patients in each group. The possibility of sensory reinnervation of flaps during surgery was not explored. Functional outcomes may have been influenced by factors unrelated to the flap itself, including tendon injuries, muscle loss, and unrecorded nerve damage at the recipient site. Although randomization of participants was done, allocation, concealment, and blinding could not be strictly adhered as the study involved a surgical procedure. However, the independent observer who assessed outcomes was blinded.

## Conclusion

From our study, it can be concluded that free flaps provide better outcome compared with pedicled flaps in terms of better aesthetic appearance and earlier functional recovery, thereby ensuring better quality of life. Earlier functional recovery following surgery, leading to quicker return to day to day activities and work, is vital in the economically productive age group, the most commonly affected age group. Although pedicled flaps remain relevant in the current microsurgical era, the results of this study show that in the presence of a dedicated microsurgical team with adequate infrastructure for performing microsurgical procedures, better reconstructive goals can be achieved with free flaps for resurfacing upper limb defects.
